# Our Contemporaries

**Published:** 1917-12

**Authors:** 


### OUR CONTEMPORARIES

Paediatrics is merged with the Medical Review of Reviews, beginning with the November issue. With the January issue of the combined journal, a special department of paediatrics will be established.

---

The Homoeopathic Envoy, Sept., quotes from a Toronto paper, to the effect that a Toronto nurse awaiting orders to go overseas, died at the Buffalo General Hospital, as the result of ''antitoxin typhoid poisoning'' from a prophylactic injection. The facts, as stated by Major N. G. Russell, are that this nurse, along with other members of Base Hospital Unit No. 23, received the regular prophylactic injection against typhoid, and paratyphoid A and B; that she had had a chronic colitis subsequently identified by the agglutination test as due to the Kruse bacillus; that the colitis did become worse after the prophylactic injection and resulted fatally; that there was nothing to indicate that the prophylactic injection was even indirectly the cause of death and that no other untoward results occurred among the considerable number vaccinated. (Note: A number of analogous cases, of the lighting up of one infection after an accidental infection with a different microorganism, or after some form of deliberate prophylactic vaccination or therapeutic use of a germ product, have been reported, especially in foreign journals. It is perfectly possible that there is a cause and effect relation. The very small proportion of such unfortunate cases in the total number of heterologous infections or deliberate introduction of some kind of germ product renders it doubtful whether these cases are or are not coincidences; whether if not coincidences, they represent a definite reaction between two heterologous specific germ products and autochthonous products of metabolism or cellular life; or whether they are instances of a general anaphylactic reaction, independent of either the existing infection or the germ products recently introduced; whether in any case, further research will enable us to determine when and where a prophylactic or therapeutic use of germ products is dangerous. The practical point is that all authorities who have investigated the problem agree that certain methods of biologic prophylaxis, as antivariolar and typhoid and paratyphoid vaccination, diphtheritic antitoxin, etc., are of the utmost value in the vast majority of instances. These claims have been supported by statistics of very large numbers of cases, the present war having, in particular, supported the experience of the Boer War and the naval and military experience of various armies in time of peace in favor of the anti-typhoid prophylaxis. Even if such cases as the one cited are directly due to prophylactic injections, they are far outbalanced by the enormous saving of life which, at least under existing war conditions, can not be ascribed to sanitary prophylaxis).

